# Yeast condensin acts as a transient intermolecular crosslinker in entangled DNA

**DOI:** 10.1093/nar/gkag044

**Published:** 2026-02-03

**Authors:** Filippo Conforto, Antonio Valdes, Willem Vanderlinden, Davide Michieletto

**Affiliations:** School of Physics and Astronomy, University of Edinburgh, Peter Guthrie Tait Road, Edinburgh EH9 3FD, United Kingdom; Chair of Biochemistry and Cell Biology, Theodor-Boveri-Institute, Julius Maximilian University of Würzburg, 97074 Würzburg, Germany; School of Physics and Astronomy, University of Edinburgh, Peter Guthrie Tait Road, Edinburgh EH9 3FD, United Kingdom; School of Physics and Astronomy, University of Edinburgh, Peter Guthrie Tait Road, Edinburgh EH9 3FD, United Kingdom; MRC Human Genetics Unit, Institute of Genetics and Cancer, University of Edinburgh, Edinburgh EH4 2XU, United Kingdom; International Institute for Sustainability with Knotted Chiral Meta Matter (WPI-SKCM^2^), Hiroshima University, Higashi-Hiroshima, Hiroshima 739-8526, Japan

## Abstract

Structural-Maintenance-of-Chromosome (SMC) complexes, such as condensins, organise the folding of chromosomes. However, their role in modulating the entanglement of DNA and chromatin is not fully understood. To address this question, we perform single-molecule and bulk characterisation of yeast condensin in entangled DNA. First, we discover that yeast condensin can proficiently bind double-stranded DNA through its hinge domain, in addition to its heads. Through bulk microrheology assays, we then discover that physiological concentrations of yeast condensin increase both the viscosity and elasticity of dense solutions of $\lambda$-DNA, suggesting that condensin acts as a crosslinker in entangled DNA, stabilising entanglements rather than resolving them and contrasting the popular theoretical picture where SMCs purely drive the formation of segregated, bottle-brush-like chromosome structures. We further discover that the presence of ATP fluidifies the solution–likely by activating loop extrusion–but does not recover the viscosity measured in the absence of protein. Finally, we show that the observed rheology can be understood by modelling SMCs as transient crosslinkers in bottle-brush-like entangled polymers. Our findings help us to understand how SMCs affect the dynamics and entanglement of genomes.

## Graphical Abstract

**Graphical Abstract:**
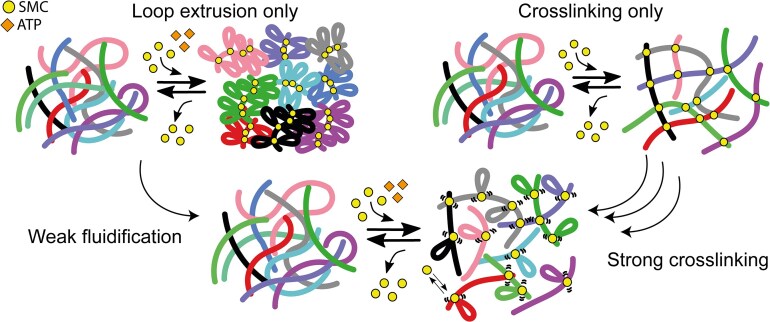

## Introduction

Among the most important processes orchestrating chromosome folding in both interphase and mitosis is the formation of loops, performed by structural-maintenance-of-chromosome (SMC) complexes, such as cohesin, condensin, and SMC5/6 [1–11]. Although these complexes perform loop extrusion *in vitro* [12–14], the extent to which loop extrusion affects genome organisation and dynamics *in vivo* is poorly understood [15–18].

Alternative models to loop extrusion are able to explain experimental observations, both *in vivo* and *in vitro*. For instance, the bridging-induced phase separation (BIPS) model can explain the formation of clusters, or condensates, of yeast cohesin in the presence of DNA [19]. The loop capture model can explain the topological trapping of a DNA plasmid by a condensin that is loaded on a tethered linear DNA [20]. Both these models rely on the fact that SMCs can ‘bridge’ inter-chromosomal DNA, i.e. can simultaneously bind two dsDNA segments that do not belong to the same DNA molecule. While there is evidence of SMC bridging for cohesin [19, 20], such evidence is less abundant for condensin. In fact, intermolecular bridging is not at all envisaged in loop extrusion models, as SMCs are envisioned to ‘reel in’ DNA *in cis* [16]. Mixed models, whereby SMCs perform an *effective* loop extrusion by bridging DNA segments, have also been proposed and can capture some puzzling evidence, for instance, the formation of Z-loops and the bypassing of large obstacles bound to DNA [21], or the observation that condensin can make steps larger than its own size [22].

SMCs are expected to have a significant impact on the dynamics of chromosomes in cells; however, it is challenging to precisely quantify this impact experimentally. The prediction from most computational and theoretical works is that loop extrusion performed by SMCs will compact [23, 24], segregate [25, 26], fluidify [27] and even unknot [28, 29] chromatin, implying that SMCs should speed up chromosome dynamics. However, indirect evidence obtained by single-particle tracking of H2B and chromosome loci suggests that rapid cohesin depletion yields a speed up of chromosome dynamics [30, 31] and nucleosome motion [32, 33]. Moreover, live-cell studies have consistently demonstrated that cohesin constrains chromatin dynamics. In fission yeast, disrupting loop factors increases locus mobility [34], a finding mirrored in mammalian mESCs upon acute cohesin depletion [35]. High-resolution tracking in human cells further revealed that this constraint operates at the nucleosome level, reducing the internal fluidity of euchromatic domains [36], implying the exact opposite of current theoretical and computational models, i.e. that cohesin slows down chromosome dynamics.

Thus, there is a clear disconnect among (i) *in vitro* single-molecule evidence displaying SMC loop extrusion, (ii) theoretical work suggesting SMC loop extrusion should drive chromosome compaction and speed up genome dynamics, and (iii) *in vivo* evidence suggesting that SMCs slow down chromosome dynamics.

In this work, we aim to bridge the gap between existing evidence and rectify this disconnect. To do this, we perform bulk and single-molecule assays on yeast condensin on *entangled* DNA *in vitro*. This is different from any previous work *in vitro* as they mostly focused on tethered DNA or dilute conditions. Instead, to understand the role of SMCs *in vivo*, we argue that we must study their behaviour in physiologically dense DNA solutions.

The key discovery of this work is that we find evidence supporting the claim that most existing computational and theoretical models are incomplete. Indeed, we observe that yeast condensin is a proficient intermolecular bridge and acts as a ‘thickening’ agent in entangled solutions of $\lambda$-DNA. Importantly, we also discovered that this ‘thickening’ is mostly loop extrusion independent. We conclude our paper by suggesting an alternative model for SMC as ‘sticky loop extruders’, which can perform both loop extrusion and intermolecular bridging, thus forming transient cross-linking in dense DNA solutions. Our results contribute to understanding the action of SMC proteins in physiologically crowded and entangled environments such as those of the cell nucleus.

## Materials and methods

### Protein expression and purification

Wild-type (WT) and Q-loop condensin holocomplexes were expressed from two 2$\mu$-based high-copy plasmids transformed into *S. cerevisiae*. Purification of holocomplexes has been performed as in Ref. [37] (see SI for more details). Expression of yeast Smc2 residues 396-792 and yeast Smc4 residues 555-951 was induced from pET-MCN vectors in bacteria. Smc2 (396-792) with an N-terminal (His)6-TEV-tag and untagged Smc4 (555-951), we co-expressed and purified by Ni-Sepharose 6FF (GE Healthcare), Resource Q (GE Healthcare), and Superdex 200 GL 10/300 column (GE Healthcare) (see SI for full details).

### Electrophoretic mobility shift assay (EMSA)

The 6-FAM labelled 50-bp dsDNA was prepared by annealing two complementary DNA oligos (Merck, 5’-6-FAM-GGATACGTAACAACGCTTATGCATCGCCGCCGCTACA TCCCTGAGCTGAC-3’; 5’-GTCAGCTCAGGGATGTAGC GGCGGCGATGCATAAGCGTTGTTACGTATCC-3’) in annealing buffer (50 mM Tris–HCl, pH 7.5, 50 mM NaCl) at a concentration of 50 $\mu$ M in a temperature gradient of 0.1 C/s from 95°C to 4°C. The EMSA reaction was prepared with a constant DNA concentration of 10 nM and the indicated concentrations of purified protein in binding buffer (50 mM Tris–HCl, pH 7.5, 50 mM KCl, 125 mM NaCl, 5 mM MgCl2, 5% Glycerol, 1 mM DTT). After 10 min incubation on ice, free DNA and DNA–protein complexes were resolved by electrophoresis for 1.5 h at 4 V/cm, on 0.75% (w/v) TAE-agarose gels at 4°C. 6-FAM labelled dsDNA was detected directly on a Typhoon FLA 9,500 scanner (GE Healthcare) with excitation at 473 nm with LPB (510LP) filter setting.

### Fluorescence polarisation

Fluorescence polarisation (FP) experiment was performed by mixing 20 nM of the 6-FAM labelled 50 bp dsDNA (see Methods EMSA) with series of protein concentrations, ranging from 0.03125 $\mu$M to 32 $\mu$M, in FP buffer (25 mM Tris-HCl pH 7.5, 100 mM NaCl, 5 mM MgCl2, 1 mM DTT, 0.05% Tween20, 0.05 mg/ml BSA). The mix was incubated for 30 min at room temperature in order to attain equilibrium. Immediately thereafter, fluorescence polarisation was recorded using 485 nm and 520 nm excitation and emission filters on a Tecan SPARK Microplate reader. The change in fluorescence polarization was then plotted as mean values of three independent replicates, and the dissociation constant was determined.

### AFM imaging

Atomic Force Microscopy (AFM) was performed on poly-L-lysine-coated mica [38]. Linear dsDNA of 500 bp was generated by PCR from pUC19 plasmid using primers 5’-AGAGCAACTCGGTCGCCGCATA (forward) and 5’-GCTTACCATCTGGCCCCAGTGC (reverse). We mixed 0.5 ng/$\mu$ L DNA and 10 nM WT condensin in aqueous buffer (50 mM Tris–HCl, pH = 7.5, 25 mM NaCl, 5 mM MgCl2, 1 mM DTT, 1 mM ATP) and incubated at room temperature for 15 s before deposition. Deposition of the sample onto poly-L-lysine-coated mica was done by drop-casting. After surface adsorption for 15 s, the sample was rinsed using milliQ water (20 mL) and subsequently dried using a gentle stream of filtered N2 gas. For imaging the sample, we used a Nanowizard 4 XP AFM (JPK, Berlin, Germany) in tapping mode; image processing was done using MountainSPIP software (see SI).

### Microrheology

For microrheology experiments, we mixed 5 $\mu$l of 500 ng/$\mu$l $\lambda$DNA with 1 $\mu$l of 2 $\mu$M yeast condensin (WT or Q), 1 $\mu$l of 10x condensin reaction buffer (Tris–HCl pH 7.5 500 mM, NaCl 250 mM, MgCl2 50 mM, DTT 10 mM), 1 $\mu$l of 10 mM ATP and 1 $\mu$l of 2 $\mu$m PEGylated polystyrene beads (Polyscience). We loaded the sample into a 100 $\mu$m thick sample chamber comprising a microscope slide, 100 $\mu$m layer of double-sided tape, and a cover slip. We recorded movies on a Nikon Eclipse Ts2 microscope with a 20x objective and an Orca Flash 4.0 CMOS camera (Hamamatsu) for 2 min at $\sim 100$ fps on a 1024x1024 field of view, resulting in about 500 tracks per condition. Particle tracking was done using trackpy, and in-house code was used to process the tracks into MSD and complex modulus in the following Ref. [39].

### Molecular dynamics simulations

Entangled DNA solutions were modelled as semiflexible Kremer-Grest linear polymers [40] with $N=500$ beads of size $\sigma = 10$ nm. The beads interact with each other via a truncated and shifted Lennard-Jones potential, and adjacent beads are connected by FENE springs. The persistence length of the polymers is $l_p = 5 \sigma = 50$ nm, and the volume fraction of the solution is around 5%. After thorough equilibration (see SI), the polymers are loaded with $N_{SMC} = \lbrace 5, 25\rbrace$ SMCs and then either let to loop extrude as in Ref. [27], or otherwise left in the loaded state to mimic conditions with no ATP. Each SMC is decorated with patches that have an attractive interaction with the DNA beads, modelled by a Morse potential with a maximum depth of $25 k_BT$, which is comparable with the heads (and hinge) binding affinity, $\Delta G \approx - k_B T \log {(k_D)}$ where $k_D \simeq 0.1$  $\mu$M. The simulation is performed in LAMMPS [41] with custom-made fixes that update the position of active loop extruders (https://git.ecdf.ed.ac.uk/taplab/smc-lammps). Specifically, our loop extrusion algorithm performs a geometry check before updating the position of the SMCs in order to preserve the topology of the system [42]. We then track the dynamics of the polymers when the SMCs are only loaded (no loops) and when allowed to make large loops via loop extrusion. In the latter case, the polymers start to resemble bottle-brushes [23, 27, 43]. At the same time, we perform Green–Kubo calculations of the stress relaxation function, i.e. we compute the autocorrelation of the off-diagonal components of the stress tensor [44], in order to obtain a measure of viscoelasticity in the system under different conditions (see SI for more details).

## Results

### Yeast condensin can form intermolecular bridges by binding dsDNA at its hinge domain

**Figure 1.**
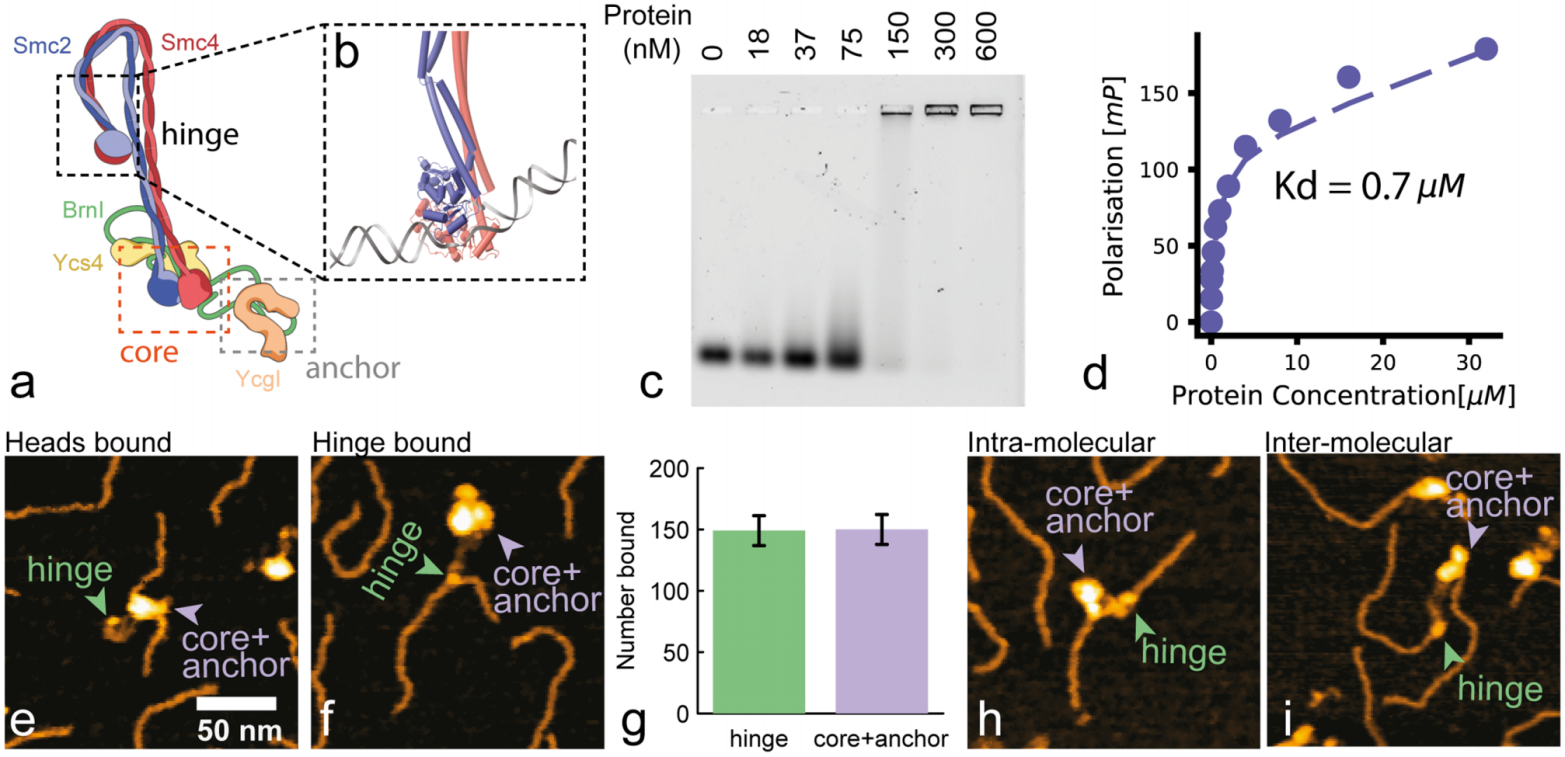
Yeast condensin proficiently binds dsDNA through its hinge domain. (**a**) Cartoon structure of yeast condensin; different domains are highlighted. (**b**) AlphaFold3 model of structural interaction between the hinge domain and a segment of dsDNA with a small ssDNA bubble in the middle. (**c**) EMSA showing significant binding of the hinge domain (SMC2:K841-L698, SMC4:Q646-F865) to a 25 bp dsDNA oligo *in vitro* with an estimated $k_D \simeq 0.075-0.15$  $\mu$M. (**d**) Fluorescence polarisation assay done with the hinge domain mixed with fluorescently-labelled 50 bp dsDNA oligo and yielding $k_D = 0.7$  $\mu$M. (**e–f**) Representative AFM topographs of (**e**) head-bound and (**f**) hinge-bound condensin–DNA complexes. Green and lilac arrowheads indicate hinges and core+anchor domains, respectively. (**g**) Quantification of relative hinge and heads bound complexes. Error bars reflect counting statistics $\sqrt{N_i}/N_{total}$. $N_{total} = 299$. (**h–i**) Representative AFM topographs of (**h**) intra-molecular and (**i**) inter-molecular condensin–DNA complexes.

First, to better understand the role of condensin in dense solutions of DNA, we decided to investigate different binding modes of yeast condensin to dsDNA. Condensin binds dsDNA through both its ‘anchor’ domain (BrnI-YcgI) [37, 45] and its ‘core’ subcomplex (SMC heads + Ycs4) [37]; however, there is no direct evidence of dsDNA binding by any other condensin domain (Fig. 1a). Thus, we sought computational evidence for an additional binding site by scanning through AlphaFold3 structures. We found a model that predicted an interaction between the SMC2/SMC4 hinge and a dsDNA oligomer containing a small ssDNA bubble (Fig. 1b). Motivated by this prediction, we performed electrophoretic mobility shift assay (EMSA) and observed a clear shift when the hinge domain (SMC2:K841-L698, SMC4:Q646-F865) was mixed with a 50 bp dsDNA segment (Fig. 1c), with an estimated binding affinity of $K_d \simeq 0.075-0.15$  $\mu$M.

This measurement was further supported by FP, where yeast condensin hinge was mixed with a fluorescently labelled dsDNA oligo, albeit we measured a larger binding constant $K_d \simeq 0.7$  $\mu$M (Fig. 1d). Interestingly, these $K_d$ values are comparable to–if not smaller than–the binding constants of the YcgI-BrnI (anchor) complex to DNA, i.e. $K_d \simeq 1.7$  $\mu$M [45] and of the ‘core’ subcomplex (SMC heads + Ycs4) $K_d \simeq 0.1 -0.2$  $\mu$M [45], both measured from *Chaetomium thermophilum*. Arguably, both EMSA and FP potentially underestimate the true $K_d$ because they employ short dsDNA oligos, which are not the natural substrate for these protein complexes; however, they convincingly demonstrate that the hinge is a proficient dsDNA binding site, potentially as good as the core/anchor subcomplex.

Motivated by these measurements, we decided to visualise dsDNA binding by the whole yeast condensin holocomplex in single-molecule experiments. We mixed yeast condensin holocomplex with a 500 bp dsDNA segment, deposited it on mica, and observed it using Atomic Force Microscopy (AFM, see Methods). We observed that yeast condensin displays different modes of binding: through its core+anchor complex, hinge, or both (Fig. 1e-f,h-i). When the holocomplex bound DNA through its core+anchor domains, we also observed a severe kink in the dsDNA molecule, in agreement with the cryo-EM structure and the ‘safety-belt’ model [37] (see Fig. 1e). On the other hand, we observed no deformation of the substrate DNA when the hinge was bound to it (see Fig. 1f).

Surprisingly, out of 299 molecules analysed, 149 were bound by the hinge and 150 bound by the core+anchor domains; when both were bound, we considered that both heads and hinge were bound. This result confirms that hinge and core+anchor domains display similar binding affinities to dsDNA. While this is broadly in line with the bulk EMSA and FP assays, it is an aspect of SMC biophysics that has been overlooked, and it is not accounted for in any of the existing models (they all start from core+anchor domains bound to DNA and an unbound hinge). Interestingly, we also observed a significant number of intra and inter-molecular bridging, whereby two segments of DNA belonging to *different* molecules are simultaneously bound by the core+anchor and hinge. This evidence suggests that yeast condensin may be a proficient ‘bridging’ protein, as observed *in vitro* for yeast cohesin [19].

Finally, we argue that while the thermodynamics of condensin domains binding to DNA may be similar, the kinetics of binding/unbinding may be very different for the two domains, e.g. due to their local flexibility. We hypothesise that the ‘safety-belt’ anchoring mechanism at the YcgI-BrnI domain may be very stable [37] (small $k_{off}$ and small $k_{on}$), whilst the kinetics at the hinge may be faster (large $k_{off}$ and large $k_{on}$). In turn, the ratio of the on/off-rates give similar equilibrium constant $K_d$. This reasoning could explain both the strong structural evidence for the ‘safety-belt’ anchoring [37] and the elusive DNA-hinge interaction as well as the potential for forming transient inter-molecular bridges, loops, and cross-links.

### Condensin acts as a transient crosslinker in entangled DNA even during loop extrusion

**Figure 2.**
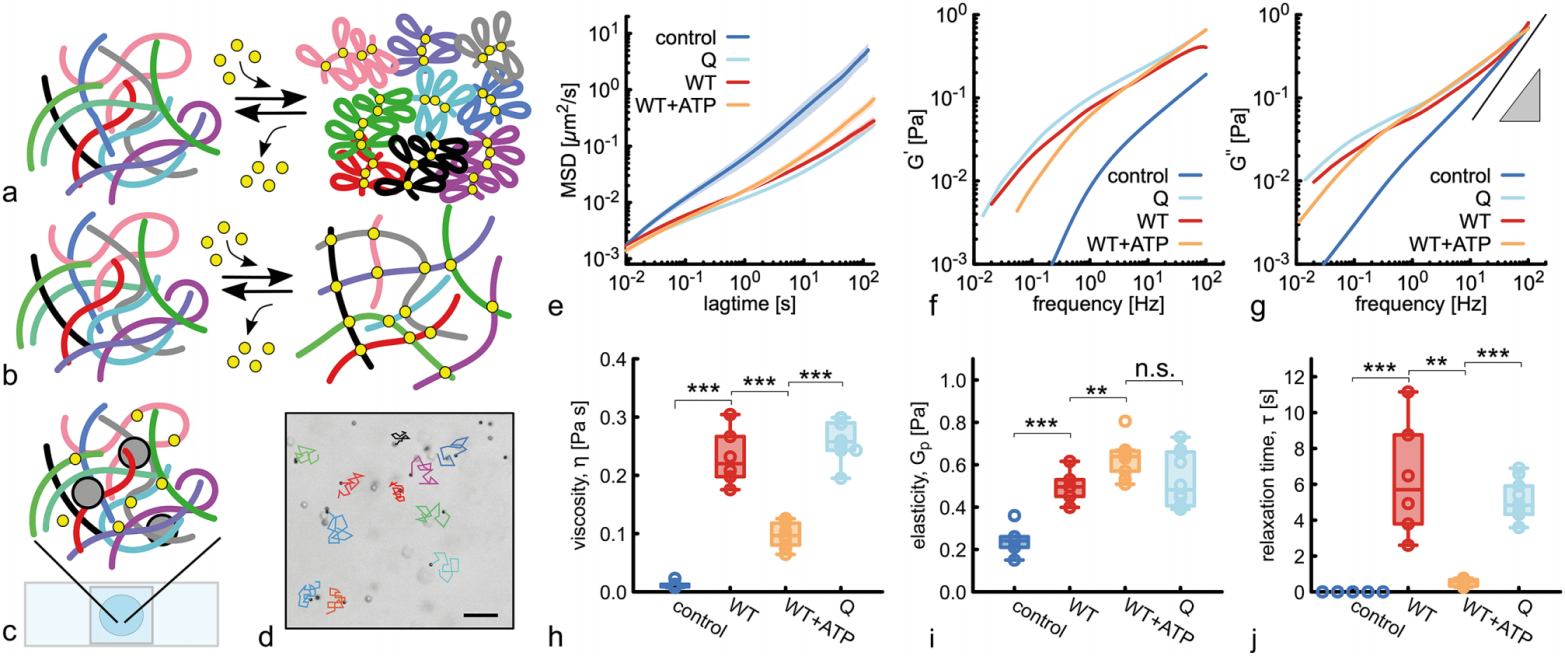
Microrheology reveals that SMCs can form intermolecular bridges in entangled DNA. (**a** and **b**) Sketches of our two hypotheses: (**a**) if condensin performed loop extrusion (intramolecular contacts only), we would expect a solution of entangled linear DNA to be converted into one made of bottle-brush-like polymers, reducing both entanglement and viscoelasticity. (**b**) If condensin performed DNA-bridging (intermolecular contacts), we would expect transient crosslinks. (**c**) The sample made of $\lambda$DNA, condensin, reaction buffer, and passive tracers is mixed, incubated, and then pipetted in a closed chamber. (**d**) Snapshot of the field of view showing the tracers and short example trajectories (scale bar 20 $\mu$m). (**e**) Mean squared displacement (MSD) of the tracer beads for wild-type yeast condensin (WT) in the presence and absence of ATP and for a catalytically dead (Q) mutant. For all samples in this figure, DNA concentration is 250 ng/$\mu$L (or 7.8 nM of $\lambda$DNA) and protein concentration is 0.2 $\mu$M, i.e. about 25 SMCs per DNA molecule (see Supplementary Fig. S5 for different concentration values). (**f** and **g**) Elastic ($G^\prime$, **f**) and viscous ($G^{\prime \prime }$, **g**) complex moduli obtained from the MSDs through the generalised Stokes Einstein relation [39]. (**h**) Zero-shear viscosity, obtained from the long-time behaviour of the MSD. P-values in the plot: $* < 0.05$, $** < 0.01$, $*** < 0.001$ . The *P*-value between WT and Q mutant is 0.13, and hence non-significant. (**i**) Elasticity $G^\prime _p$ obtained from the elastic modulus measured at 100 Hz. (**j**) Relaxation time $\tau _R$, obtained as the inverse of the smallest frequency at which $G^\prime$ and $G^{\prime \prime }$ intersect.

To understand the effect of SMC intermolecular bridging observed in the previous section, we decided to assess the effect of SMCs on the rheology of entangled DNA. We hypothesised that if condensin was mainly performing intra-molecular loop extrusion, we would observe a significant decrease in entanglements and a consequent fluidification of the solution, i.e. a *decrease* in viscosity. This hypothesis is in line with current models of SMCs on chromosomes and DNA, where loop extrusion is envisaged to drive the formation of bottle-brush-like structures [10, 23–25, 27, 43, 46, 47]. On the other hand, intermolecular bridging would give rise to gel-like networks, whereby DNA-DNA entanglements would be stabilised by condensin bridges. These entangled networks of DNA are expected to display larger viscosity and elasticity than equally dense DNA solutions without SMC (Fig. 2a,b).

To quantitatively measure condensin effect on the viscoelasticity of DNA solutions, we prepared samples of entangled $\lambda$-DNA (48’502 bp) at around 12 times the overlap concentration ($c = 250$ ng/$\mu$l $= 7.8$ nM, $c^{*} = 20$ ng/$\mu$l [48, 49]), and mixed it with 0.2 $\mu$M of either wild type (WT) yeast condensin or a catalytically dead (Q-loop) mutant that cannot perform loop extrusion [37]. These conditions represent a dense, entangled solution of long monodisperse DNA whereby each polymer has, on average, 10-20 SMCs loaded onto it (or 1 SMC every 5 kbp). We also included 2 $\mu$m-sized PEG-passivated polystyrene tracer beads (we obtained similar results with different bead sizes, see Supplementary Fig. S4) and adjusted buffer conditions to those used to observe loop extrusion in single-molecule assays [14, 37]. After incubation at 37$^\circ$C for 5 minutes, we added 1mM ATP and loaded 5 $\mu$l of sample onto a chamber made of a glass slide and coverslip, kept apart by a 100 $\mu$m spacer, and visualised it under an inverted microscope (Fig. 2c). We then performed microrheology, i.e. recorded videos of the passive tracers moving in the solution and extracted their mean squared displacement (MSD) $\delta ^2 r(t) = \langle [ \boldsymbol {r}(t+t_0) - \boldsymbol {r}(t_0) ]^2\rangle$, where the average is performed over beads, initial times $t_0$, sample location, and at least three independent replicates (see Fig. 2e).

According to most current models, SMCs should compact DNA by performing loop extrusion and thus decrease the viscosity of the entangled solution [23, 27]. In our experiment, this fluidification would manifest itself as an increase in the effective diffusion coefficient of the tracer beads and an absence of subdiffusive behaviour [49]. On the contrary, we observed the opposite: a significant decrease in the mobility of the beads and an increase in their subdiffusive regime for both WT and Q-loop condensin and in both presence and absence of ATP (Fig. 2e and Supplementary Fig. S3). To quantify the elastic and viscous response of the fluid at different timescales, we transformed the MSDs into elastic ($G^\prime (\omega )$) and viscous ($G^{\prime \prime }(\omega )$) complex moduli via the generalised Stokes-Einstein relation [39, 50]. In Fig. 2f-g, one can appreciate that the presence of WT and Q-loop condensin significantly affects the shape of $G^\prime (\omega )$ and $G^{\prime \prime }(\omega )$. More specifically, the control displays a purely viscous behaviour with little sign of inflection in $G^{\prime \prime }(\omega )$; on the contrary, the samples with SMCs display at least one intersection between the two complex moduli (see SI). This entails that the fluid’s response is elastic-dominated at short timescales (large $\omega$) and liquid-dominated at long timescales (small $\omega$). Finally, we also observe that SMCs induce a significant increase in both elasticity and viscosity of the samples across all frequencies.

We compute the zero-shear viscosity of the samples $\eta = k_B T/(3 \pi a D)$, where $a = 2$  $\mu$m is the size of the beads and $D$ the large-time diffusion coefficient obtained from the MSDs. We note that adding SMCs induces a 20-30-fold increase in viscosity in all samples, with the increase being more pronounced for WT and Q-loop mutant (Fig. 2h). Interestingly, the sample with WT SMC and ATP displays the smallest change ($\sim 10$-fold), which may point to a fluidification effect of loop extrusion.

We can also compute the large-$\omega$ elasticity $G^{\prime }_p$ and relaxation time $\tau _R$ of these viscoelastic fluids by evaluating $G^{\prime }_p=G^\prime (\omega =\textrm {100~Hz})$ and $G^\prime (1/\tau _R) = G^{\prime \prime }(1/\tau _R)$, respectively. The former ($G^{\prime }_p$, Fig. 2i) suggests that the short-time elastic behaviour is significantly stiffer for SMC samples, regardless of whether there is ATP or not. Despite this observation, all samples display $G^{\prime }_p < 1$ Pa, implying that they are very soft. On the other hand, the latter ($\tau _R$, Fig. 2j) suggests that samples with WT condensin and ATP behave like liquids on shorter timescales (smaller $\tau _R$) than the ones without ATP or with the Q-loop mutant, which remain solid-dominated for longer times, up to tens of seconds.

Our observations suggest that SMCs may form transient cross-links between DNA molecules that are proximal in 3D space. Further, our results suggest that WT in the absence of ATP behaves similarly to the Q-loop mutant. This suggests that both proteins bind DNA similarly and that the Q-loop mutation does not affect the crosslinking ability of SMC. However, in the presence of ATP, we observe a significant ‘fluidification’, which we argue is an effect of loop extrusion. However, we find that this fluidification is not strong enough to fully counteract the transient SMC-mediated crosslinking.

### Coarse-grained MD simulations of ‘sticky’ SMCs capture the behaviour seen in bulk and single-molecule assays

**Figure 3.**
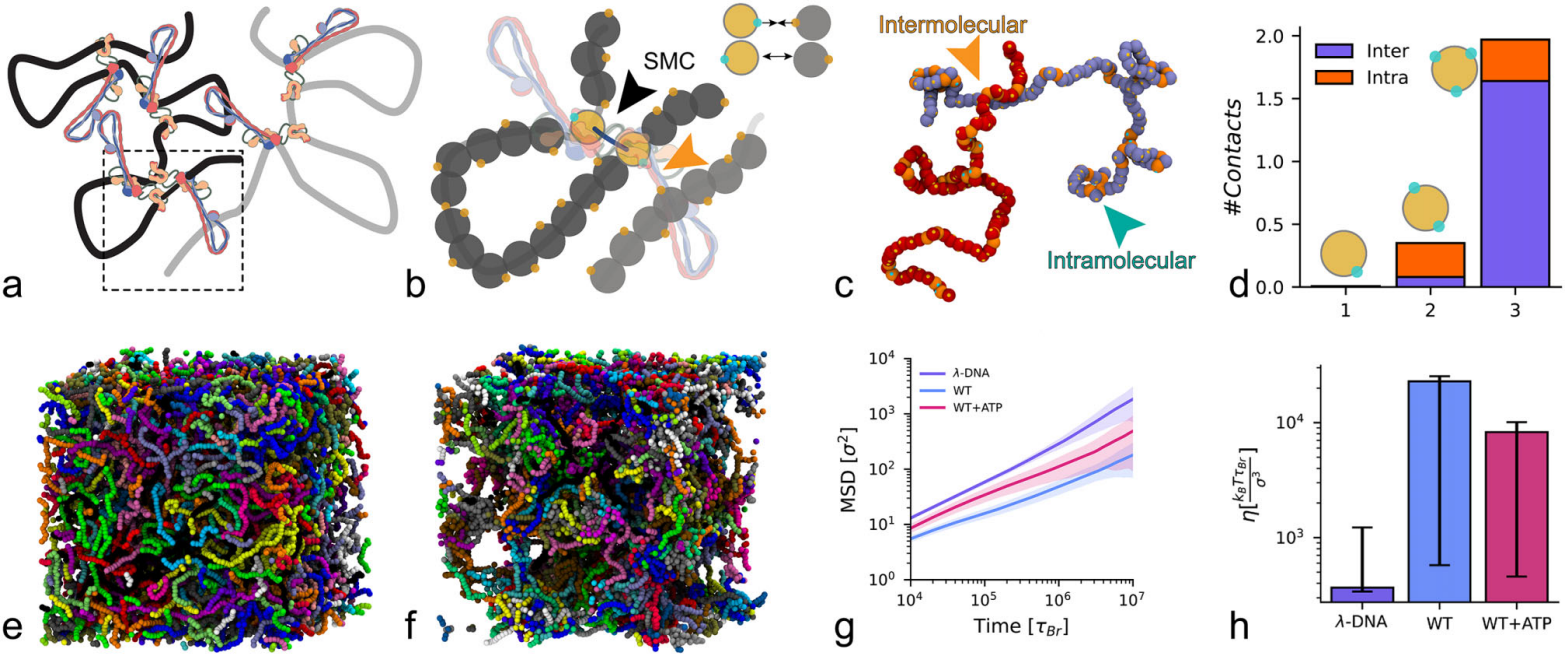
MD simulations of sticky SMCs model the thickening. (**a**) Sketch of DNA with loops formed by SMCs. (**b**) Bead-spring polymer modelling of the region in the dashed box showing the correspondence between patches (cyan) and the hinge domain. (**c**) Snapshot from simulations, highlighting intramolecular and intermolecular interactions stabilised by the patches. (**d**) Average number of contacts as a function of the number of patches on the beads. (**e** and **f**) Snapshots of the simulation box in two cases: (**e**) in equilibrium with no SMC and (**f**) after loading 50 sticky SMCs per polymer and allowing them to extrude loops. (**g)**. Average Mean Squared Displacement (MSD) of the polymers’ centre of mass (standard deviation shaded) for the control case ($\lambda$-DNA) compared with the cases with SMC but no extrusion (WT) and the case with SMC allowed to extrude loops (WT+ATP). (**h**) Viscosity computed from the stress-relaxation function (see SI) for the three cases in (**g**). Notice that with ATP, the system is more fluid, in line with experiments.

Motivated by the observations in the previous Sections we decided to test a simple coarse-grained model of ‘sticky’ loop extruders. Specifically, we performed Molecular Dynamics (MD) simulations of entangled linear DNA under the action of SMCs that display small patches that can bind to DNA polymers (see Fig. 3a-b). Briefly, we modelled DNA molecules as Kremer-Grest bead-spring polymers [40] at fixed density and in the entangled regime, corresponding to 5% volume fraction. After equilibrating the system, we randomly loaded, on average, 5 SMCs per polymer and allowed them to form both DNA loops *in cis* and inter-molecular bridges through their patches (Fig. 3a-c). Our SMC model is different from most models in the literature, as we allow the SMCs to do both, form intra/inter molecular bridges through their patches and also form loops through extrusion, whilst preserving the polymer topology (see SI for full details).

To account for the formation of SMC clusters, we also explored the effect of having multiple DNA-binding sites on each SMC bead: on average, less than one contact per SMC complex was seen for patches per SMC bead, while 2 contacts (mostly inter-molecular) were seen for $N_p = 3$ patches per SMC bead (Fig. 3d). This implies that only one-third of all SMC patches were bound to DNA at any one time and corresponds to the case in which there are two clustered, or stacked, SMCs per loop. In this scenario, the two SMCs are bound to the same DNA by their core+anchor domains, and have two ‘free’ hinges that can make inter or intramolecular contacts with other DNA segments (see Fig. 3a-b).

We observed that once the SMCs are loaded, the system qualitatively displayed remarkable clustering (see snapshots Fig. 3e-f). By computing the mean squared displacement of the centre of mass of the polymers, i.e. $\delta ^2 r (t) = \langle \left[ \boldsymbol {r}_{CM}(t + t_0) - \boldsymbol {r}_{CM}(t) \right]^2 \rangle$, we discovered that they also displayed a significantly slower dynamics (Fig. 3g-h). More specifically, to compare our simulations with the experiments, we performed two sets of simulations: (i) SMCs are bound to DNA and cannot loop extrude (case with no ATP or Q-loop mutant in experiments), and (ii) SMCs are allowed to extrude loops (case with ATP in experiments). The MSDs displayed in Fig. 3g show that the dynamics of the polymers in the presence of extrusion are faster than the no-extrusion case, in agreement with experiments. We argue that this result is explained by the fact that in the latter case the polymers were forming bottle-brush-like organisations that reduced the overall entanglements and sped up their dynamics [23, 27]. We further computed the stress relaxation function $G(t)$ through the autocorrelation of the out-of-diagonal components of the stress tensor [44, 51] (see Supplementary Fig. S6) and computed the zero-shear viscosity of the simulated fluid. In line with the microrheology, the DNA solution with SMCs that cannot perform loop extrusion displays a 20-fold increase in viscosity. Allowing the SMC to loop extrusion only reduces the viscosity by a small ($\sim$2-fold) factor, but the dominant effect remains the transient ‘gelling’ of the DNA entanglements.

## Discussion and conclusions

In summary, in this paper, we have provided experimental and computational evidence that SMCs, and specifically yeast condensin, can stabilise inter-molecular interactions in solutions of dense DNA.

First, we used biochemical assays and AFM to uncover that the hinge domain of yeast condensin is a proficient dsDNA binding site (Fig. 1a-d). Unexpectedly, we observed that it binds as strongly as condensin heads, which are well-known DNA binding sites from structural studies [37, 45]. We also note that although AlphaFold3 predicted the hinge domain binding to a ssDNA bubble, our experimental data clearly show that it can bind both ss and dsDNA (see EMSA and FP in Fig. 1c-d, e-f, and Supplementary Figs S1 and S2). Interestingly, we also note that previous experiments suggested that condensin stepping can undertwist DNA, which is consistent with the formation of a small ssDNA bubble (5-6 bp $\Delta Tw \simeq -0.5$) [52]. It is therefore tempting to hypothesise that SMC binding and stepping may itself induce the formation of a single-stranded bubble on dsDNA. Finally, by performing AFM imaging, we report visual evidence that SMCs can simultaneously bind dsDNA through heads and hinge domains, forming both intra and inter-molecular contacts (Fig. 1h-i).

In light of this evidence, we reasoned that if yeast condensin were to be introduced in a dense and entangled solution of DNA, it would mediate inter-molecular bridges. However, this hypothesis would be at odds with a large fraction of the current loop extrusion models, which posit that SMCs mostly perform intra-chain loop extrusion and no bridging, leading to mostly unentangled, bottle-brush-like chromosome structures [2, 4, 16, 46]. We therefore decided to test the action of yeast condensin on entangled DNA using microrheology. Specifically, we performed experiments with $\lambda$-DNA at volume fraction comparable to that of DNA in yeast cells, i.e. $12 \textrm {Mbp} \times [\pi (0.34 \textrm {nm/bp}) (2.5 \textrm {nm})^2]/(4 \mu m^3) \simeq 2\%$ , or about 3 mg/ml [49]. We decided to use 0.25 mg/ml of $\lambda$-DNA at low ionic conditions, 25 mM NaCl, which yields an effectively larger DNA diameter of about 12 nm [53]; in turn, these conditions yield an entangled solution of $\lambda$-DNA at an effective $\sim 1\%$ volume fraction.

We reasoned that if pure intra-chain loop extrusion was the dominant mode of action of SMCs, we would observe a speed up of the dynamics of entangled DNA solutions, leading to a so-called ‘thinning’ of the solution’s viscosity (Fig. 2a-b). In contrast, we consistently observed that adding yeast condensin to our solution of entangled DNA led to so-called ‘thickening’, i.e. an increase in the solution’s viscosity and mirroring a slowdown of DNA dynamics (Fig. 2e-g). This effect cannot be attributed to the mere presence of additional protein in the solution because (i) we observe an increase in elasticity, implying the formation of DNA crosslinks, and (ii) we observed thinning with different proteins (e.g. with IHF in Ref. [49]). Our microrheology data also reveal that adding ATP partially recovers the DNA dynamics. Since we excluded that this effect is due to ATP itself (see Supplementary Fig. S3), we thus concluded that the partial fluidification observed in the presence of ATP and WT protein is likely due to ATP-driven loop extrusion partially counter-acting intermolecular crosslinking (Fig. 2h). Finally, we discover that the SMC Q-loop mutant, despite its inability to hydrolise ATP, can also form intermolecular crosslinks, as strong as the wild type condensin (Fig. 2i-j).

To connect the rheology and single-molecule observations, we concluded this work by performing MD simulations where we modelled SMCs as ‘sticky’ proteins that can both stabilise dynamic intermolecular cross-links and form loops. This computational model yielded results in line with what was observed experimentally (Fig. 3a-b, g-h).

**Figure 4.**
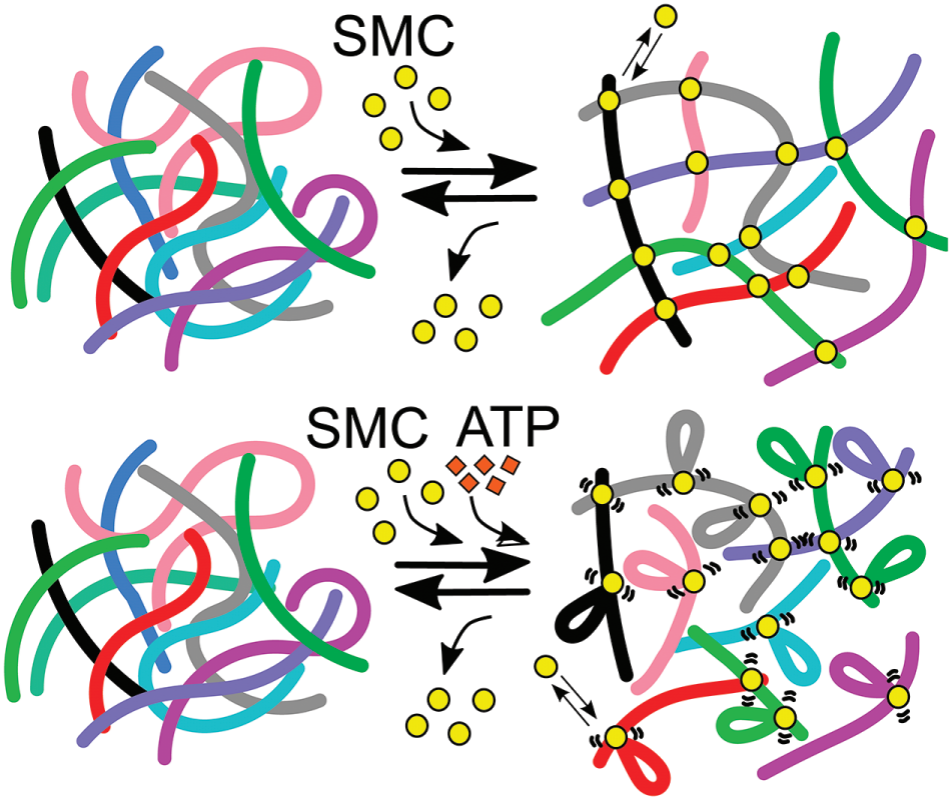
SMCs act as transient intermolecular crosslinkers that can perform intramolecular loop extrusion in the presence of ATP. (top) SMCs loaded on DNA form transient intermolecular bridges by simultaneously binding dsDNA molecules through their heads and hinge domains. (bottom) ATP-driven loop extrusion competes with intermolecular bridging and speeds up DNA dynamics.

In light of this, we therefore argue that in our *in vitro* experiments, SMCs do not exclusively form intra-chain loops (as predicted by loop extrusion models), but also form inter-molecular transient crosslinks, in turn affecting the solution’s entanglements and viscoelasticity (Fig. 4). Our model is in line with the ‘bridging-induced’ phase separation behaviour observed in yeast cohesin [19, 54], and the evidence that condensin can slow down chromatin dynamics in mitosis and interphase [32, 55]; it is also in agreement with the role of condensin in sequestering repetitive DNA in the nucleolus [26]. By having identified the hinge as an additional dsDNA binding domain, our model of SMC acting as both intermolecular crosslinker and intramolecular loop extruder can naturally explain other models, e.g. the ‘loop capture’ [18, 56] and ‘inter-molecular loop-extrusion’ [21, 57] models.

The overall picture is that SMCs form a dynamic and reversible mesh of weakly cross-linked polymers, whereby the cross-links themselves may be mobile if SMCs are performing loop extrusion (Fig. 4). In fact, we argue that this system is physically similar to so-called slide-ring gels, where polymers in solution thread through ring-like molecules that can form crosslinks and slide along the chains [58].

Finally, we should highlight that our microrheology experiments are the first *in vitro* evidence that condensin forms intermolecular, transient crosslinks in entangled DNA solutions. Rheology measurements on DNA solutions offer a clear quantification of the impact of SMCs on entangled DNA. Since our experiments are performed at around $\sim$ 1% volume fraction, we believe that they are closer to physiological concentrations than current single molecule assays, e.g. DNA tethering or optical and magnetic tweezers [14, 59]. The effects uncovered in this work are therefore expected to be physiologically relevant and could in fact explain the puzzling observations from single molecule tracking *in vivo*, whereby depletion of cohesin typically induces a speed up of chromatin dynamics in interphase [30, 31, 55] and depletion of condensin speeds up nucleosome dynamics during metaphase [60]. At the same time, our work can explain the role of condensin in stiffening chromosomes through bridging [61].

To conclude, we argue that SMCs’ role in regulating genome organisation and dynamics may be more multifaceted and complex than previously thought. Our experiments suggest that SMC intermolecular bridging is a dominant mechanism of action on entangled DNA and that intramolecular loop extrusion may potentially make a minor contribution. Additionally, the relative weight of these contributions may be modulated across the cell cycle by partner proteins. In the future, we argue that microrheology will be an ideal assay to test the presence of partner proteins and additional cofactors in a physiologically relevant condition of DNA density.

## Supplementary Material

gkag044_Supplemental_File

## Data Availability

The code has been deposited in Zenodo: https://doi.org/10.5281/zenodo.17098515.
