# Supplementary material for: Yeast condensin acts as a transient intermolecular crosslinker in entangled DNA
Source: Nucleic Acids Res. 2026 Feb 3;54(3):gkag044. doi: 10.1093/nar/gkag044 (PMC12865461; doi:10.1093/nar/gkag044)
Supplement: gkag044_Supplemental_File [file gkag044_supplemental_file.pdf]

Supplementary Information: Yeast condensin acts as a transient intermolecular crosslinker in entangled DNA

Filippo Conforto,[1] Antonio Valdes,[2] Willem Vanderlinden,[1] and Davide Michieletto[1,3,4]

[1]*School of Physics and Astronomy, University of Edinburgh, Peter Guthrie Tait Road, Edinburgh, EH9 3FD, UK*

[2]*Department of Biochemistry and Cell Biology, Julius Maximilian University of Würzburg, 97074 Würzburg, Germany*

[3]*MRC Human Genetics Unit, Institute of Genetics and Cancer, University of Edinburgh, Edinburgh EH4 2XU, UK*

[4]*International Institute for Sustainability with Knotted Chiral Meta Matter (WPI-SKCM^2), Hiroshima University, Higashi-Hiroshima, Hiroshima 739-8526, Japan*

MATERIALS AND METHODS

Microrheology

For microrheology experiments, we mix 5 μl of λDNA at 500 ng/μl, with 1 μl of 2.1 μM yeast condensin, 1 μl of 10x condensin reaction buffer (Tris-HCl Ph 7.5 500 mM, NaCl 250 mM, MgCl2 50 mM, DTT 10 mM), 1 μl or 10 mM ATP and 1 μl of 2 μm PEGylated polystyrene beads (Polyscience). We load the sample into a 100 μm thick sample chamber comprising a microscope slide, 100 μm layer of double-sided tape and a cover slip. We perform experiments using an Nikon Eclipse Ts2 microscope with a 60x objective and Orca Flash 4.0 CMOS camera (Hamamatsu). We record a series of movies for 2 minutes at $\sim$ 100 fps on a 1024x1024 field of view, resulting in about 500 tracks per condition.

We use TrackPy and custom-written particle-tracking codes (in Python and C++) to extract the trajectories of the diffusing beads and measure the time-averaged MSDs of the diffusing particles as a function of lag time t. We note that while the tracked trajectories are in 2D, because the samples are isotropic, we average the x and y direction as if they were independent walks, such that all MSDs shown and used to determine viscosities, are determined from the average of the MSDs in the x and y directions. We compute diffusion coefficients D via linear fits to the MSDs according to $MSD = (2d)Dt$ (with d = 1, because the x and y directions from 2D tracking are averaged together). From D, we compute the zero-shear viscosity ν using the Stokes–Einstein equation $\eta = k_B T/(3\pi D a)$ with a the diameter of the particles. We also compute the elastic G' and viscous G'' moduli by employing the generalised Stokes-Einstein relation [1, 2].

AFM sample preparation, imaging, and image processing

As a substrate for sample deposition, we prepared poly-L-lysine-coated mica by drop-casting 20 μL poly-L-lysine (Merck; 0.01% w/v in autoclaved milliQ water) on freshly cleaved muscovite mica (SPI Supplies) for 30 s and subsequently rinsing the surface with 20 mL of milliQ water before drying with a gentle stream of filtered N2 gas [3]. Linear DNA (500 bp, generated by PCR from pUC19 plasmid using primers 5'-AGAGCAACTCGGTCGCCGCATA (forward) and 5'-GCTTACCATCTGGCCCCAGTGC (reverse)) was mixed at final concentrations of 0.5 ng/μL DNA and 10 nM WT condensin in aqueous buffer (50 mM Tris-HCl pH = 7.5, 25 mM NaCl, 5 mM MgCl2, 1mM DTT, 1mM ATP) and incubated at room temperature for 15 s before deposition. Deposition of the sample onto poly-L-lysine coated mica was done by dropcasting. After surface adsorption for 15 s, the sample was rinsed using milliQ water (20 mL) and subsequently dried using a gentle stream of filtered N2 gas. For atomic force microscopy imaging, we used a Nanowizard 4 XP AFM (JPK, Berlin, Germany) in tapping mode with silicon tips (FASTSCAN-A; drive frequency, 1,400 kHz; Bruker) over fields of view of 6 $\times$ 6 μm at 4,096 $\times$ 4,096 pixels and captured at line rates of 3 Hz. AFM image processing of the raw topographic data was done using MountainSPIP software (v10, Digital Surf) included plane-fitting with a 3rd degree polynomial, and line-by-line correction with a 4th degree polynomial.

Electrophoretic mobility shift assay (EMSA)

The 6-FAM labeled 50-bp dsDNA was prepared by annealing two complementary DNA oligos (Merck, 5'-6-FAM-GGATACGTAACAACGCTTATGCATCGCCGCCGCTACATCCCTGAGCTGAC-3'; 5'-GTCAGCTCAGGGATGTAGCGGCGGCGATGCATAAGCGTTGTTACGTATCC-3') in annealing buffer (50 mM Tris-HCl pH 7.5, 50 mM NaCl) at a concentration of 50 μM in a temperature gradient of 0.1 C/s from 95°C to 4°C. The EMSA reaction was prepared with a constant DNA concentration of 10 nM, either with dsDNA or ssDNA (Merck, 5'-6-FAM-CCACTCCGAC), and the indicated concentrations of purified protein in binding buffer (50 mM Tris-HCl pH 7.5, 50 mM KCl, 125 mM NaCl, 5mM MgCl2, 5% Glycerol, 1 mM DTT). After 10 min incubation on ice, free DNA and DNA-protein complexes were

resolved by electrophoresis for 1.5 hr at 4 V/cm, on 0.75% (w/v) TAE-agarose gels at 4°C. 6-FAM labeled dsDNA was detected directly on a Typhoon FLA 9,500 scanner (GE Healthcare) with excitation at 473 nm with LPB (510LP) filter setting.

Fluorescence Polarisation

Fluorescence polarization (FP) experiment was performed by mixing 20 nM of the 6-FAM labeled 50 bp dsDNA or 10mer ssDNA (see Methods EMSA) with series of protein concentrations, ranging from 0.03125 μM to 32 μM, in FP buffer (25 mM Tris-HCl pH 7.5, 100 mM NaCl, 5 mM MgCl2, 1 mM DTT, 0.05% Tween20, 0.05 mg/ml BSA). The mix was incubated for 30 min at room temperature in order to attain equilibrium. Immediately thereafter, fluorescence polarization was recorded using 485 nm and 520 nm excitation and emission filter on a Tecan SPARK Microplate reader. The change in fluorescence polarization was then plotted as mean values of three independent replicates and the dissociation constant determined.

Purification of Saccharomyces cerevisiae condensin holocomplex

All five subunits, of wild-type and ATP mutants condensin complexes, were expressed from two 2μ-based high copy plasmids under the control of galactose-inducible promoters transformed into *S. cerevisiae.* One plasmid contained pGAL10-YCS4 pGAL1-YCG1 TRP1 and the other pGAL7-SMC4-StrepII3 pGAL1-SMC2 pGAL1-BRN1-His12-HA3 URA3 or their ATP mutant derivates. Cultures were maintained at 30°C in −Trp–Ura medium with 2 % (w/v) D-glucose, transferred to −Trp–Ura medium with 2 % (w/v) raffinose for 6 h and overexpression was induced by addition of 2 % (w/v) D-galactose for 16 h. Cell lysates were prepared in a FreezerMill (Spex) in lysis buffer (50 mM Tris-HCl pH 7.5, 200 mM NaCl, 5 % (v/v) glycerol, 5 mM β-mercaptoethanol, 20 mM imidazole) supplemented with cOmplete EDTA-free protease inhibitor mix (cOm–EDTA, Roche). The lysate was cleared by centrifugation at 45,000 3 gmax and loaded onto Ni-Sepharose 6FF (GE Healthcare). After washing with 30–40 column volumes (cv) lysis buffer, proteins were eluted in 7–10 cv elution buffer (lysis buffer plus 300 mM imidazole). The eluate was supplemented with 0.01 % (v/v) Tween-20, 1 mM EDTA and 0.2 mM PMSF, incubated for about 16 h with Strep-Tactin Superflow high-capacity resin (2-1208-010, IBA) and eluted with St-elution buffer (50 mM Tris-HCl pH 7.5, 200 mM NaCl, 5 % (v/v) glycerol, 1 mM dithiothreitol (DTT) containing 10 mM desthiobiotin (D1411, Merck). The eluate was concentrated by ultracentrifugation before size-exclusion chromatography on a Superose 6 increase 10/300 column (Cytiva) pre-equilibrated in SEC-buffer (25 mM TRIS-HCl pH 7.5, 500 mM NaCl, 1 mM DTT). Peak fractions were pooled and concentrated by ultrafiltration (Vivaspin 30,000 MWCO, Sartorius).

Purification of Saccharomyces cerevisiae condensin hinge

The DNA fragments encoding yeast Smc2 residues 396-792 and yeast Smc4 residues 555-951 were inserted into a pET MCN vector by standard PCR-based cloning methods. Smc2 (396-792) with an N-terminal (His)6-TEV-tag and Smc4 (555-951) without a tag were co-expressed in the Escherichia coli Rosetta (DE3) pLysS (Merck) grown at 18°C in 2 X TY medium. Cells were lysed by sonication at 4oC in lysis buffer (50 mM TRIS-HCl pH 7.5, 200 mM NaCl, 20 mM imidazole, 5 mM b-mercaptoethanol) containing cOmplete protease inhibitor cocktail tablets without EDTA (cOm–EDTA, Roche). The lysate was cleared by centrifugation at 45,000 3 gmax and loaded onto Ni-Sepharose 6FF (GE Healthcare). After washing with 30–40 column volumes (cv) lysis buffer, proteins were eluted in 7–10 cv elution buffer (lysis buffer plus 300 mM imidazole). The eluate was dialyzed overnight in dialysis buffer (25 mM TRIS-HCl pH 7.5, 200 mM NaCl, 1 mM DTT) at 4°C. The dialyzed eluate was diluted with low-salt buffer (25 mM TRIS-HCl pH 7.5, 100 mM NaCl, 1 mM DTT) to a final salt con- centration of 150 mM NaCl and loaded onto a 6 mL RESOURCE Q (GE Healthcare) anion exchange column pre-equilibrated with low- salt buffer. After washing with 3–5 cv low-salt buffer, proteins were eluted by increasing NaCl concentrations to 1 M in a linear gradient of 60 mL. Peak fractions were pooled and loaded onto a Superdex 200 GL 10/300 column (GE Healthcare) equilibrated in SEC-buffer (25 mM TRIS-HCl pH 7.5, 500 mM NaCl, 1 mM DTT). Peak fractions were pooled and concentrated by ultrafiltration (Vivaspin 30,000 MWCO, Sartorius).

EMSA and FP demostrate that the hinge domain can bind ssDNA

Through additional EMSA assays and Fluorescence Polarisation (FP) experiments we showed that the hinge domain of yeast condensin is able to bind both dsDNA and ssDNA. Figure S1 displays an EMSA assay done with a 10 mer ssDNA oligo, while Figure S2 shows a FP assay done with the same ssDNA oligo. Both experiments show significant binding of the hinge domain to ssDNA in vitro. The dissociation constant k_D is estimated to be 0.48 from FP, similar to the value reported in the main text for dsDNA ($k_D = 0.7$).

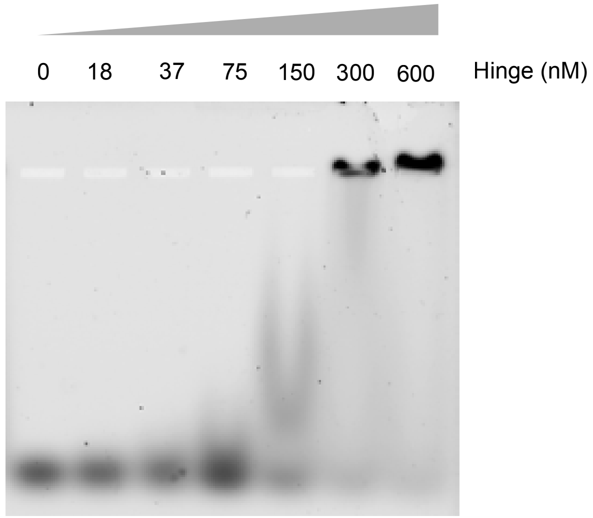

FIG. S1. EMSA showing significant binding of the hinge domain (SMC2:K841-L698, SMC4:Q646-F865) to a 10 mer ssDNA oligo in vitro.

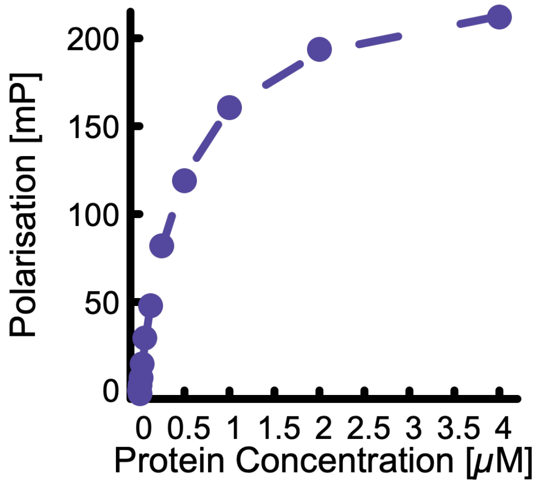

FIG. S2. Fluorescence polarisation assay done with the hinge domain mixed with fluorescently-labelled ssDNA oligo of 10 mer length. Estimated $K_d = 0.48$ μM.

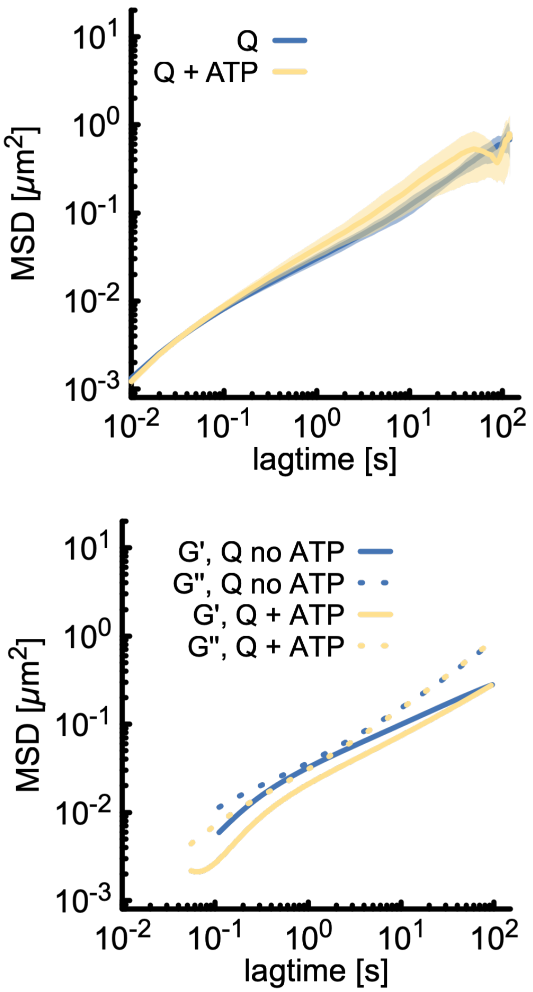

FIG. S3. (Top) Mean squared displacement (MSD) of 2μm tracer beads for a solution of dense DNA (prepared as in the main text) containing Q-loop yeast mutant, in presence and absence of ATP. (bottom) G' and G'' for the samples including the Q mutant with and without ATP display similar behaviours.

Additional microrheology experiments

We performed additional microrheology experiments to assess the effect of ATP and size of passive tracer on the outcome of these measurements. First, we performed microrheology of a solution of λ-DNA, as in the main text, in the presence of yeast condensin Q-loop mutant (able to bind DNA but not to hydrolise ATP and therefore not able to loop extrude). We find that the MSDs of the tracers (here 2 μm particles) are similar in both presence and absence of ATP (see Fig. S3). This results confirms that ATP by itself does not significantly affect the rheology of the solution or the activity of the Q-loop mutant.

Second, we performed microrheology experiments with different bead sizes to assess the role of bead size in our measurements. It is well known that while bigger tracers are better at capturing the mesoscopic rheology of the solution, small enough beads will diffuse through the entanglements and will therefore not capture mesoscale rheology [4]. Since we expect the mesh size in our experiments to be around $\simeq 0.5$ μm [5], we decided to test passive tracers with diameter > 1 μm. We thus repeated our microrheology measurements using 1, 2 and 3 μm sized beads; we also tested different conditions: e.g. no protein and with or without ATP, to measure potential effects of Mg chelation by ATP. Because we work at low

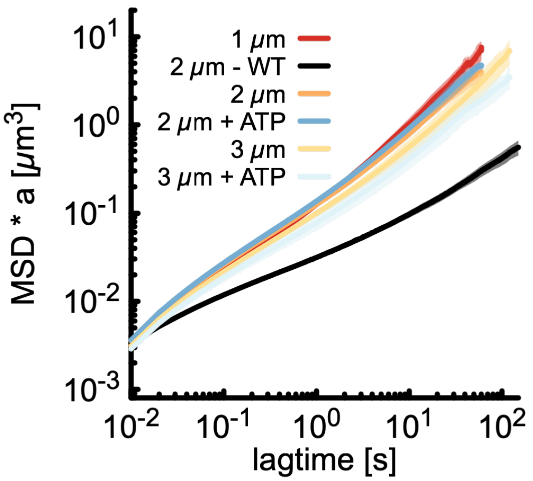

FIG. S4. Mean squared displacement (MSD) multiplied by the bead size (a) computed for tracers of different sizes (1 μm, 2 μm and 3 μm) in presence or absence of ATP (no protein) and in the presence of protein and no ATP (WT).

ATP concentration (1 mM) and MgCl concentration (5 mM), we do not expect significant chelation effects overall. Indeed, in fig. S4 we plot the MSD multiplied by the bead size (MSD $\times$ a) as a function of lag-time for experiments done with different beads and different ATP conditions. As one can appreciate, the MSD $\times$ a measured with 1 μm bead is overlapping with the one measured with the 2 μm bead in both presence and absence of ATP. The MSD $\times$ a measured with the 3 μm bead is slightly slower than expected, however the impact of bead size is not enough to significantly affect the MSD slow down observed in the presence of SMC protein (see black curve 2 μm - WT in the figure).

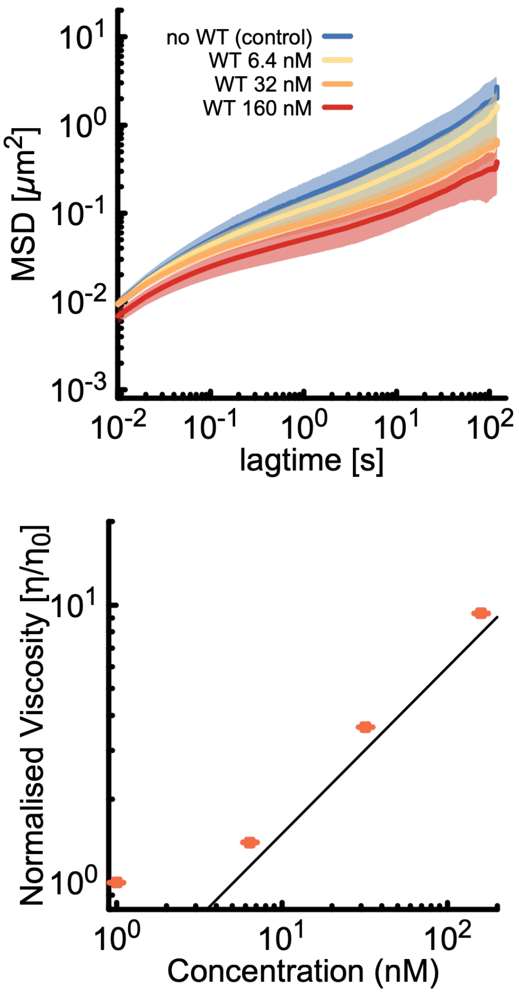

FIG. S5. (Top) Mean squared displacement (MSD) of tracer beads for solutions of dense λ-DNA prepared as in the main text with different concentration of WT condensin. (Bottom) Viscosity of the solution at different SMC concentration normalised by the control (no protein) viscosity. The black line is a guide for the eye, scaling as $c^{0.6}$.

Microrheology at different condensin concentration

In this section we report microrheology experiments performed at lower condensin concentrations. The MSDs, shown in Fig. S5, display an expected behaviour, at smaller protein concentration the mobility of the tracer increases, eventually matching the behaviour seen in absence of protein. The normalised viscosity scales with the concentration of SMC as $\sim c^{0.6}$.

Molecular Dynamics Simulations

We model entangled DNA as semiflexible Kremer-Grest linear polymers [6] with $N = 500$ beads of size σ. The beads interact with each other via a truncated and shifted Lennard-Jones potential,

$$U_{\rm LJ}(r) = \begin{cases} 4\epsilon\left[\left(\dfrac{\sigma}{r}\right)^{12} - \left(\dfrac{\sigma}{r}\right)^{6} + \dfrac{1}{4}\right] & r \le r_c \\ 0 & r > r_c \end{cases} , \quad (1)$$

where r denotes the separation between the beads and the cut-off $r_c = 2^{1/6}\sigma$ is chosen so that only the repulsive part of the potential is used. Nearest-neighbour monomers along the contour of the chains are connected by finitely extensible nonlinear elastic (FENE) springs as,

$$U_{\rm FENE+LJ}(r) = \begin{cases} -0.5kR_0^2 \ln\left(1 - (\frac{r}{R_0})^2\right) + U_{\rm LJ} & r \le R_0 \\ \infty & r > R_0 \end{cases} , \quad (2)$$

where $k = 30\epsilon/\sigma^2$ is the spring constant and $R_0 = 1.5\sigma$ is the maximum extension of the elastic FENE bond.

This choice of potentials and parameters is essential to preclude thermally-driven strand crossings and therefore ensures that the global topology is preserved at all times [6, 7]. Finally, we add bending rigidity via a Kratky-Porod potential, $U_{\text{bend}}(\theta) = k_\theta \left(1 - \cos\theta\right)$, where θ is the angle formed between consecutive bonds and $k_\theta = 5k_BT$ is the bending constant, thus yielding a persistence length $l_p = 5\sigma$, corresponding to 50 nm in our grained model. We chose these parameters to facilitate the comparison with *in vitro* experiments, i.e. to model the behaviour of naked DNA at physiological salt condition resulting in a screening length of 10 nm and a persistence length of 50 nm. Each bead's motion is then evolved via the Langevin equation

$$m\frac{dv_i}{dt} = -\gamma v_i - \nabla U + \sqrt{2k_BT\gamma_i}\eta \tag{3}$$

along each Cartesian component. Here, γ is the friction coefficient, m the mass of the bead, U the sum of the potentials acting on bead i and $\sqrt{2k_BT\gamma}\eta$ a noise term that obeys the fluctuation-dissipation theorem, thus respecting the formula

$$\langle \eta_i^\alpha(t)\eta_j^\beta(s)\rangle = \delta(t-s)\delta_{ij}\delta_{\alpha\beta}$$

along each Cartesian component (Greek letters). The numerical evolution of the Langevin equation is done with a velocity-Verlet scheme with $dt = 0.01\tau_{LJ}$ with $\tau_{LJ} = \tau_{Br} = \sigma\sqrt{m/\epsilon}$ in LAMMPS [8].

Patches are placed at a distance of 0.5σ from the core of the beads, on the surface of each bead. Their movement follow the one of the beads they belong to. The sticky ends interaction, responsible for bridging, is modelled by a Morse potential:

$$U_m(r) = \epsilon_m \left[e^{-2\alpha_0(r-r_0)} - 2e^{-\alpha_0(r-r_0)}\right]$$

for $r < R_c$. Here, r represents the distance between patches of two adjacent nanostars, $r_0 = 0$ is their equilibrium distance and $R_c = 0.2\sigma$ is the cut-off distance of attraction. The amplitude of the potential is set to $\epsilon_m = 25.0k_BT$ and $\alpha_0 = 14\sigma^{-1}$ controls the width of the potential. These parameters were chosen to ensure that during the simulation each bead can hybridize with any (but only one) of the other beads. Since the hard-sphere repulsion of beads (set by the LJ potential) covers a radius of 0.56σ, we expect hybridized patches to be at a minimum distance of 0.12σ.

The system simulated consist in a solution of 50 linear polymers having size 500 in a cubic box of side length 64, achieving a monomer density of ∼10%, equivalent to a volume fraction of $\phi = 0.05$.

Modelling Loop Extrusion

Our loop extrusion model was inspired by previous works [9, 10] which were also implemented into the LAMMPS engine. In these models, loops are formed by temporary bonds joining two beads, thus generating closed rings emerging from the polymer backbone. Loop extrusion is then achieved by shifting each bond to the adjacent beads on both sides of the bond. However, these model allow non-physical extrusion since bond movement is performed irrespectively of the distance between selected beads. This is often possible thanks to the use of unbounded harmonic bonds, allowing large distances between the loop ends and possibly leading third segments to pass through the bonded segments. We argue that this non-physical feature should be avoided as we expect SMC complexes to block possible strand passages in between their ends, and that extrusion should take into account the geometry and topology of the DNA molecule [11]. Moreover, according to recent observations [12] extrusion has proven to be asymmetric, thus enforcing the need of pulling of only one of the two sides of the bond. Therefore, we developed a customized version of a LAMMPS "fix" module publicly available at `https://git.ecdf.ed.ac.uk/taplab/smc-lammps` and used the version v3_05_07_2024 for all the included simulations. In practice, we implement loop extrusion by initialising a given number of SMCs by choosing random triplets of beads belonging to the polymers in solution. This provides each polymer with a total number of bound SMCs on average equal to n_{SMC}. However, to neglect the presence of unextruded polymers we place at least one SMC on every polymer. We define the extrusion direction at the moment of the first displacement, and let the bond move and extrude a loop. Specifically, we attempt extrusion steps with a fixed frequency f_{att}, chosen at the beginning of the simulation. On top of that, we define a success probability f_{prob} for the extrusion step, that adds up to the geometry check. Then, the distance between new SMCs' heads is computed, and the step is accepted only if its value is smaller than a fixed cut-off $r < 1.2\sigma$. Each SMC attempts an effective step with frequency $f_{eff} = f_{att}f_{prob}$, in turn slowing down the actual extrusion speed along the polymer because of conformational entropy. If during the extrusion process two SMCs meet on one end, extrusion stops. Consequently, extrusion runs for each SMC until the extruding end neighbours another SMC's end or reaches the polymer ends. In the simulations we changed the value of $f_{eff} = 1 \cdot 10^{-3}\,\tau_{Br}^{-1}$ to account for different extrusion speed of the SMC complexes. Specifically we set $f_{eff} = 1 \cdot 10^{-3}\,\tau_{Br}^{-1}$, to obtain a realistic extrusion speed and evolved our simulation for the equivalent of $3 \cdot 10^6\,\tau_{Br}$ to realise loops of different lengths. The latter scenario corresponds to the system defined as WT+ATP in the main text, while SMC binding and no extrusion corresponds to the case defined as WT.

To prevent integration errors related to the bond length, we initially model LEFs with a harmonic bond,

with potential

$$U_{\text{harm}}(r) = A(r - R_0)\,, \tag{4}$$

where $A = 100$ and $R_0 = 1.1\sigma$. After the first step, such bond is replaced by a FENE bond with $k = 10$ and $R_0 = 1.7\sigma$ (see Eq. 2). We choose a larger maximum extension for the elastic FENE bond and a softer spring constant to avoid bond breaking caused by sudden movement of the bonds during extrusion. Bridging interaction is achieved by displacing uniformly (at angle of 180° from each other if two patches, at an angle of 120° from each other on a plane, if three patches) patchy beads on each of the beads involved in the simulation.

Modelling SMCs with different valence

We can model different valence (number of interactions) by changing the number of patches on the SMC beads, as displayed in Fig. 3d in the main text. With $N_p = 3$ we observed that each SMC on average makes two contacts, whilst a smaller number of patches greatly reduces the number of intermolecular contacts.

Green-Kubo calculation

The stress-relaxation modulus G(t) is calculated as

$$G(t) = \frac{V}{3k_bT}\sum_{\alpha\neq\beta}\bar{P}_{\alpha\beta}(0)\bar{P}_{\alpha\beta}(t)\,, \tag{5}$$

where ($\bar{P}_{\alpha\beta} = \bar{P}_{xy}$ and $\bar{P}_{xz}$ and $\bar{P}_{yz}$) represents the off-diagonal components of the stress tensor. Specifically, we get those components as

$$\bar{P}_{\alpha\beta}(t) = \frac{1}{t_{avg}}\sum_{\Delta t=-\frac{t_{avg}}{2}+1}^{t_{avg}} P_{\alpha\beta}(t+\Delta t)\,, \tag{6}$$

$$P_{\alpha\beta}(t) = \frac{1}{V}\left(\sum_{k=1}^{NM} m_k v_k^\alpha v_k^\beta + \frac{1}{2}\sum_{k=1}^{NM}\sum_{l=1}^{NM} F_{kl}^\alpha r_{kl}^\beta\right), \tag{7}$$

where N is the number of beads per polymer, M the number of polymers, V the box volume, m_k the mass of the k-th bead, v_k the speed of the k-th bead, F_{kl} the force between the k-th and the l-th bead and r_{kl} their distance. $P_{\alpha\beta}$ is then averaged over a time t_{avg} [13]. The autocorrelation was computed using the multiple-tau correlator method described in reference [14] and implemented in LAMMPS with the fix ave/correlate/long command. This method makes sure that the systematic error of the multiple-tau correlator to be always below the level of the statistical error of a typical simulation (see LAMMPS documentation). The viscosity η of the system is then obtained by integrating $G(t)$ as,

$$\eta = \int_0^{t\to\infty} G(t)dt$$

Given that our simulation are run for a finite time, the computed viscosity represents a lower bound for the true value. Such value is then obtained in simulation units $\frac{k_BT\tau_{Br}}{\sigma^3}$ where $\tau_{Br} = \frac{3\pi\eta_s\sigma^3}{k_BT} = 2.3\mu s$.

To account for the noisy values appearing on large timestep values we model the behaviour of G(t) as a stretched exponential at large times. Specifically, we define $G(t) \approx ae^{\left(-\frac{t}{\tau}\right)^b}$, and we fit a,$\tau$,b to approximate the exponential decay, starting at an arbitrarily found point, denoted as t_e. The viscosity is then obtained by numerical integration up to t_e, while the stretched exponential contribution is obtained by computing $\int_{t_e}^{\infty} ae^{\left(-\frac{t}{\tau}\right)^b} = \frac{a\tau}{b}\Gamma\left(\frac{1}{b},(\frac{t_e}{\tau})^b\right)$, where $\Gamma(a,z)$ is the upper generalised gamma function. The sum between these two terms returns the viscosity estimate. The fitting range is set around the length-scale at which the stress-relaxation function show the exponential decay behaviour. When this is not possible, in cases in which the relaxation timescale is longer than the duration of the simulation itself we fit the function with a power law up to $10^7\tau_{Br}$ and use the integrated value as a lower bound estimate of the viscosity. The errors shown in Fig. 3 are defined as the range between lower bound of viscosity defined by integrating numerically up to the the fitting range and the upper bound obtained by integrating numerically up to the relaxation timescale.

The Green-Kubo (GK) measurements are done in equilibrium in all the scenarios considered in the main text.

Measuring contact density

The contact densities displayed in Fig. 3d are obtained by measuring the average number of neighbours each SMC has in the simulation over a time equal to $1 \cdot 10^6\tau_{Br}$. We define as neighbours the beads whose patches are found to be distant from the SMC beads less than a cutoff defined as 0.2σ. By dividing it by the number of SMC in solution we get the average number of contacts per SMC, with an error that is below 1%. Despite this consistency in the number of contacts, by fitting the exponential decay of the autocorrelation function we were able to estimate the average active time of the attractive morse between two different patches. Effectively this interaction allows the formation of temporary bridges of average duration equal to $73 \pm 2\,\tau_{Br}$ for the system correspondent to WT, and values around $\approx 80\tau_{Br}$ for all the tested systems. This means that the interaction modelled through this patchy system are effectively transient but can lead to semi-permanent links

between the polymers.

The density of SMCs affects the rheology of the system

Additionally, we tested the effect of different density of SMCs in our solution of polymers. We explored systems in which SMC were distributed such to have in average 50 SMC per polymer, which is 10 times larger than the systems considered in the main text. The viscoelasticity is then affected by the increase in the number of contacts between the polymers, especially the intermolecular ones favour the elastic behaviour of the solution, as visible in the two curves representing the WT behaviour.

Surprisingly, allowing loop extrusion in such a dense system seems to favour the decrease in mobility - oppositely to what it has been reported in the main text. We argue that this effect is due to the accumulation of SMCs along the polymer. Because of the higher density and thus smaller loop size, on average shorter than the case with 5 SMC per polymer, the SMCs form clusters and thus lead to effectively stronger intermolecular bridging. As displayed in Fig. S6b this clustering results in a very marked entanglement plateau in the WT+ATP case, yielding in enhanced elasticity.

Modelling SMCs with different valence

We can model different valence (number of interactions) by changing the number of patches on the SMC beads, as displayed in Fig. 3d in the main text. With $N_p = 3$ we observed that each SMC on average makes two contacts, whilst a smaller number of patches greatly reduces the number of intermolecular contacts.

As displayed in Fig. S7a, as we increase the number of patches, the MSD of the polymers' center of mass becomes more elastic at short times. Interestingly, with $Np = 0$ and $Np = 1$ we observe an increase in mobility of the polymers, in line with what we have seen in our previous work [15]. Oppositely, $Np = 2$ and $Np = 3$ show significant subdiffusion; similarly, the stress relaxation function in Fig. S7b shows an increase in the elastic plateau for $Np = 2$ and $Np = 3$, and the absence of an exponential decay following the elastic plateau. The integration of these curves through the fit at long times display a similar behaviour, with viscosities larger than the control for both $Np = 2$ and $Np = 3$.

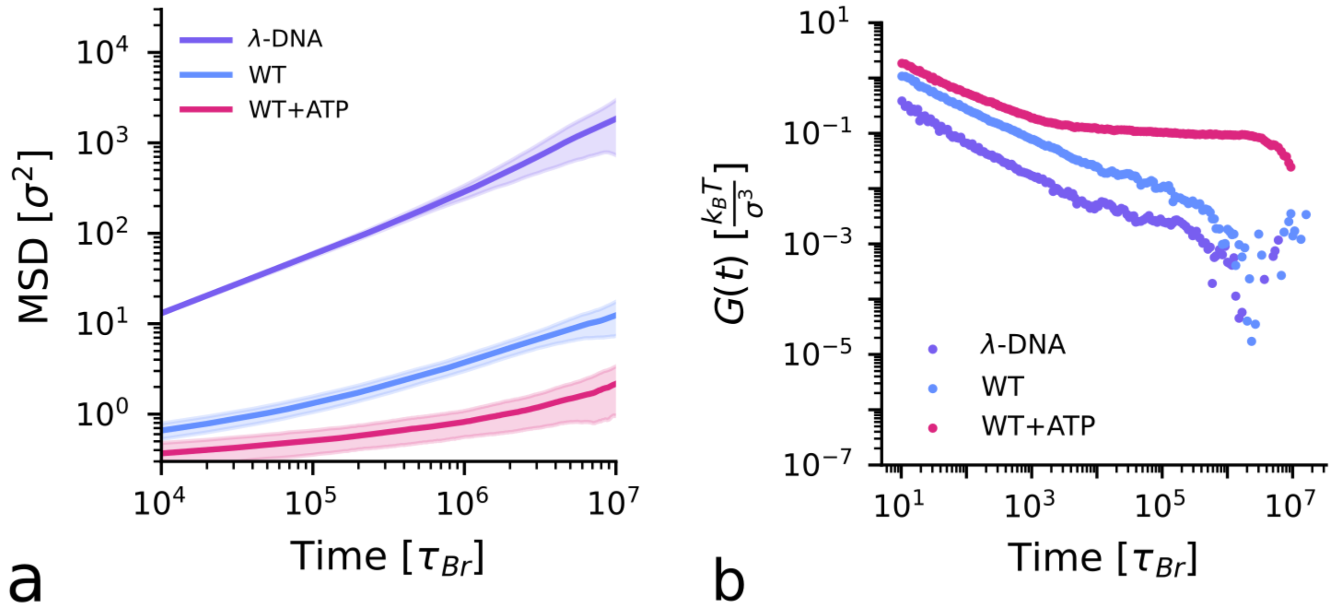

FIG. S6. **High density case: 50 SMC per polymer. a.** Average Mean Squared Displacement (MSD) of the polymers' center of mass (standard deviation shaded) for the control case (λ-DNA) compared with the cases with SMC but no extrusion (WT) and SMC with extrusion (WT+ATP). **b.** Stress-relaxation function ($G(t)$) for the control case (λ-DNA) compared with the cases with SMC but no extrusion (WT) and SMC with extrusion (WT+ATP).

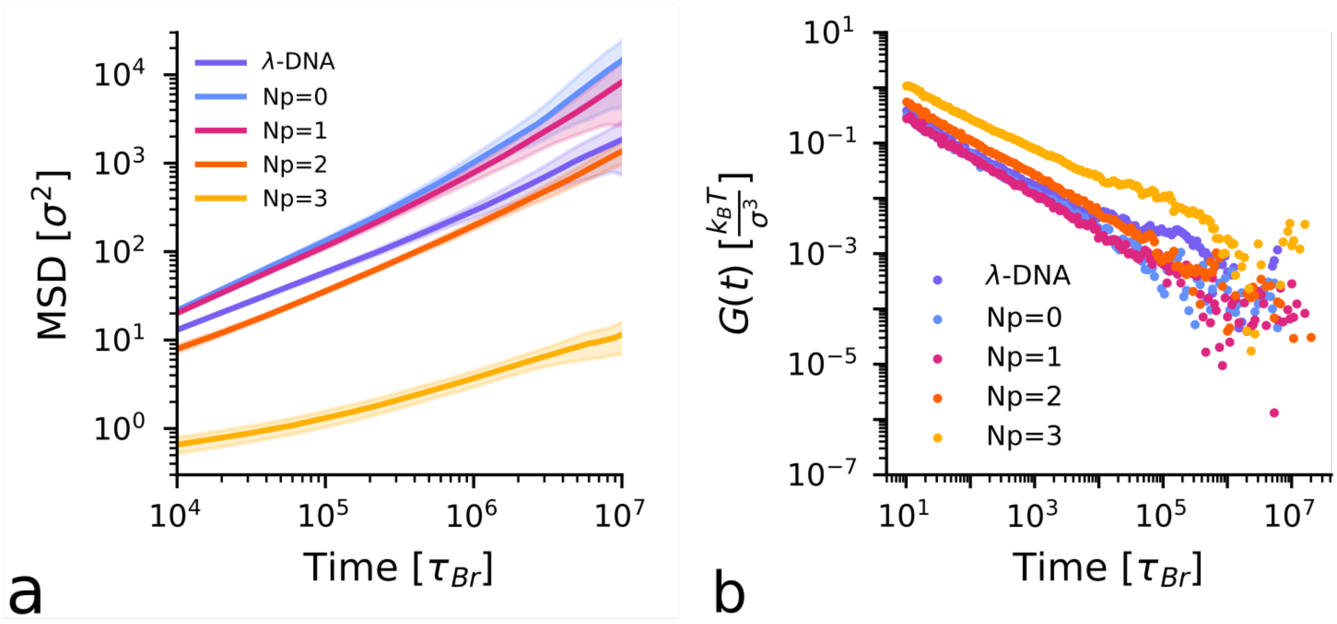

FIG. S7. **Effect of number of patches on the solution dynamics. a.** Average Mean Squared Displacement (MSD) of the polymers' center of mass (standard deviation shaded) for the control case (λ-DNA) compared with the cases with SMC (no extrusion) and three different N_p. **b.** Stress-relaxation function (G(t)) of the solution of polymers for the control case (λ-DNA) compared with the cases with SMC (no extrusion) and three different N_p. In both these plots, the system has 50 SMCs per polymer.

[1] T. G. Mason, Rheologica Acta **39**, 371 (2000).

[2] J. Harnett, S. Weir, and D. Michieletto, Soft Matter **20**, 3980 (2024).

[3] W. Vanderlinden, J. Lipfert, J. Demeulemeester, Z. Debyser, and S. De Feyter, Nanoscale **6**, 4611 (2014).

[4] D. T. Chen, E. R. Weeks, J. C. Crocker, M. F. Islam, R. Verma, J. Gruber, A. J. Levine, T. C. Lubensky, and A. G. Yodh, Physical Review Letters **90**, 108301 (2003).

[5] Y. A. G. Fosado, J. Howard, S. Weir, A. Noy, M. C. Leake, and D. Michieletto, Physical Review Letters **130**, 058203 (2023).

[6] K. Kremer and G. S. Grest, The Journal of Chemical Physics **92**, 5057 (1990).

[7] L. Tubiana, G. P. Alexander, A. Barbensi, D. Buck, J. H. Cartwright, M. Chwastyk, M. Cieplak, I. Coluzza, S. Čopar, D. J. Craik, M. Di Stefano, R. Everaers, P. F. Faísca, F. Ferrari, A. Giacometti, D. Goundaroulis, E. Haglund, Y. M. Hou, N. Ilieva, S. E. Jackson, A. Japaridze, N. Kaplan, A. R. Klotz, H. Li, C. N. Likos, E. Locatelli, T. López-León, T. Machon, C. Micheletti, D. Michieletto, A. Niemi, W. Niemyska, S. Niewieczerzal, F. Nitti, E. Orlandini, S. Pasquali, A. P. Perlinska, R. Podgornik, R. Potestio, N. M. Pugno, M. Ravnik, R. Ricca, C. M. Rohwer, A. Rosa, J. Smrek, A. Souslov, A. Stasiak, D. Steer, J. Sułkowska, P. Sułkowski, D. W. L. Sumners, C. Svaneborg, P. Szymczak, T. Tarenzi, R. Travasso, P. Virnau, D. Vlassopoulos, P. Ziherl, and S. Žumer, Physics Reports **1075**, 1 (2024).

[8] S. Plimpton, J. Comp. Phys. **117**, 1 (1995).

[9] G. Fudenberg, M. Imakaev, C. Lu, A. Goloborodko, N. Abdennur, and L. A. Mirny, Cell Reports **15**, 2038 (2016).

[10] A. Goloborodko, M. V. Imakaev, J. F. Marko, and L. Mirny, eLife **5**, e14864 (2016).

[11] E. Orlandini, D. Marenduzzo, and D. Michieletto, Proceedings of the National Academy of Sciences **116**, 8149 (2019).

[12] B. Pradhan, A. Pinto, T. Kanno, D. Tetiker, M. D. Baaske, E. Cutt, C. Chatzicharlampous, H. Schüler, A. Deep, K. D. Corbett, L. Aragon, P. Virnau, C. Björkegren, and E. Kim, bioRxiv (2024), 10.1101/2024.09.12.612694.

[13] W. B. Lee and K. Kremer, Macromolecules **42**, 6270 (2009).

[14] J. Ramírez, S. K. Sukumaran, B. Vorselaars, and A. E. Likhtman, J . Chem. Phys. **133**, 154103 (2010).

[15] F. Conforto, Y. Gutierrez Fosado, and D. Michieletto, Phys. Rev. Res. **6**, 033160 (2024).
